# Establishment of a Basement Membrane-Related Prognosis Model and Characterization of Tumor Microenvironment Infiltration in Acute Myeloid Leukemia

**DOI:** 10.7150/jca.108041

**Published:** 2025-01-13

**Authors:** Zongsi Zhu, Yuedong Zhao, Ping Li

**Affiliations:** Department of Hematology, Tongji Hospital, Tongji University School of Medicine, Shanghai, China.

## Abstract

**Background:** Basement membrane is a special component of extracellular matrix of epithelial and endothelial tissues, which can maintain their normal morphologies and functions. It can also participate in tumor progression and affect tumor treatment. However, the roles of basement membrane-related genes (BMGs) in acute myeloid leukemia (AML) remain unknown.

**Material and methods:** We downloaded the data of AML and normal samples from TCGA, GTEx, and GEO. Then, we performed bioinformatics analysis to identify differential BMGs. We calculated the risk score of the training cohort and divided it into two risk groups. In addition, we also introduced external cohorts, serving as validation cohorts, to estimate the accuracy of risk score. A nomogram was established based on the risk score and clinicopathological characteristics to predict the prognosis. Based on BMGs, AML patients of TCGA were clustered into 2 subtypes. To investigate the biological features and the association between immune cells and TME, we utilized GSVA to assess pathway enrichment and ssGSEA to quantify the levels of immune cell infiltration across samples.

**Results:** We obtained 3 differential BMGs between AML and normal samples. The training cohort was divided into high- and low-risk groups based on the risk score. The Kaplan-Meier survival analysis indicated that the two groups had significant differences. The nomogram could be used to predict the survival outcomes of AML patients. Based on the clustering result, we found significant differences between the two gene clusters. Sankey's diagram suggested that cluster B was associated with the high-risk group and poor prognosis. GSVA analysis showed that cluster B was also related to the upregulation of intercellular and intracellular signal transduction pathways. In TME, resting mast cells, follicular helper T cells, and plasma cells decreased while monocytes increased in the high-risk group. In addition, the high-risk group was more sensitive to BTK and AKT inhibitors.

**Conclusion:** Our study indicated that the nomogram model of BMGs could predict the prognosis of AML patients. Meanwhile, BMGs were correlated with immune TME in AML. A correct and comprehensive assessment of the mechanisms of BMGs in individuals will help guide more effective treatment.

## Introduction

Acute myeloid leukemia (AML) is a hematological malignancy characterized by the abnormal proliferation of immature myeloblasts. It can infiltrate bone marrow and impair hematopoiesis 1. It can be subdivided into several types by blast elements and proportion. Chemotherapy, such as anthracycline and cytarabine, is a classical therapy for AML and can prolong the survival time. Moreover, the emergence of many novel therapies, such as chimeric antigen receptor (CAR) T cell therapy and molecular targeted therapy also brings benefits to AML patients 2. Despite advancements in therapeutic strategies, AML continues to present significant challenges due to its heterogeneous nature, high relapse rates, and resistance to conventional treatments, necessitating ongoing research to develop more effective and targeted therapies.

Basement membrane (BM) is an important component of cell-adherent extracellular matrices (ECM), which covers the basal surface of epithelial tissues and surrounds the deep tissues 3. It protected tissues against disruptive physical stresses and provided an interactive interface between the extracellular environment and cells 4. Matrix metalloproteinases participation in the metastatic process and their diagnostic and therapeutic applications in cancer. It is also able to control cell polarity, adhesion, and migration 5. Previous research has shown that BM is involved in tumor invasion in many aspects. Proteases are the enzymes that selectively degrade peptide bonds between amino acids of BM 6. Cancer cells can secret proteases, especially matrix metalloproteinases (MMP), to degrade collagens and increase metastasis 7, 8. In addition, some emerging studies reveal that physical mechanisms may also be involved in tumor invasion of BM 9. Early studies have indicated that tumor cells will adopt a non-proteolytic migration mode when proteases are inhibited 10. Proteases have been linked with immune cells since immune cells frequently used them to form gaps to cross vascular BM and enter into blood circulation during the inflammation process 11. Then, the migration of immune cells led to remodeling and enlargement of these BM gaps. The restored gaps become slightly larger than their original size after a crossing immune cell crossed, which is beneficial for other cells to pass through. This mechanism is convenient for tumor metastasis 12. It has been indicated that, in the protease-independent BM invasion model, tumor cells can cross BM via the activities of immune cells. Therefore, it is suggested that BM could be disrupted by immune cells, which may facilitate the migration of tumor cells.

Previous studies have clarified that BM is closely related to a variety of human epithelial cancers and is a pivotal prognostic factor promoting the invasion of tumors. Once the tumor cells break the BM barrier, invade the surrounding stromal tissues, and spread through blood and lymphatic vessels, the overall survival time will rapidly decrease 13. Siegel *et al.* indicated that if breast carcinoma cells remained localized, the 5-year survival rate of patients could reach 99%. However, once cancer cells invaded the BM and the surrounding deep tissues, the 5-year survival rate decreased to 85%. Moreover, the 5-year survival rate of breast cancer dropped to 27% when there was distant metastasis 14. Similarly, the disruption of BM integrity is an indispensable factor in prostate, skin, gastric and colorectal cancers 15-18.

In hematology, the bone marrow microenvironment regulates the proliferation, differentiation, and migration of hematopoietic stem cells (HSCs) 19. ECM membrane allows the stem cell to interact with stromal cells and promotes HSCs migration and differentiation 20. Besides solid tumors, hematopoietic cells in the marrow microenvironment can also secrete proteases that play various roles in the hematological system. Previous studies have confirmed that proteases also play important roles in hematopoietic differentiation and progression of hematological diseases 21. The specific functions were involved in tumor's invasion and angiogenesis 22. As the main gelatinases of proteases, MMP-9 and MMP-2 were detected in normal bone marrow cells and megakaryocytes (MKs). Meanwhile, MMP-2 is detected in erythroblasts. They can release growth factors and cytokines from the ECM membrane, leading to stem cell-stromal cell interactions that promote HSCs migration and differentiation 20. In addition, MMPs are potential markers for diagnosis of hematological diseases. MMP-9 could be used to predict survival outcomes in patients with early-stage chronic lymphocytic leukemia (CLL) in hematological malignancies 23. The serum levels of MMP-2 and MMP-9 are reported to be abnormal in both myelodysplastic syndrome and AML 24.

In this study, we explored the expression pattern of BM-related genes (BMGs) in AML, understanding the role of BM in the prognosis of AML. First, we collected clinical information and transcriptome data of AML and normal samples from public databases. Then, we obtained BMGs by using bioinformatics and constructed a nomogram model to predict the prognosis of AML patients. Moreover, we also attempted to clarify the relationship between BMGs and TME of AML. Finally, we investigated the drug sensitivity in two risk groups, which may provide new therapeutic options.

## Methodology

### Data acquisition and arrangement

The transcriptome data of AML were downloaded from The Cancer Genome Atlas (TCGA) and The Gene Expression Omnibus (GEO). To ensure the reliability and accuracy of survival results, we excluded samples with incomplete clinical data and without survival data. We obtained 142 AML samples from TCGA as the training cohort and 564 AML samples from GEO (ID: GSE12417, GSE37642) as the validation cohort. In addition, we selected 337 normal blood samples from The Genotype-Tissue Expression (GTEx) as the control group. Raw RNA-seq data were normalized to TPM values using the “edgeR” package to adjust for sequencing depth and gene length. Batch effects between datasets were corrected using the ComBat function from the “sva” package, with batch labels corresponding to the dataset sources. PCA analysis confirmed effective normalization and batch effect removal, as samples grouped according to biological characteristics post-correction.

We obtained the somatic mutation information and copy number variation (CNV) data of AML patients from UCSC Xena. We used the “maftools” package to analyze the mutation frequencies and the results were plotted with the oncoplot waterfall plot. All analyses in this study were performed by R (version 4.4) with R Bioconductor packages and Perl software.

### Identification of differential BMGs

We obtained 222 BM-related genes (Table S1) from the published articles 4. And we compared the samples expression of TCGA AML with normal samples and determined 20 differentially-expressed BM genes by the “edgeR” package 25. Then, we merged the survival data (survival time and status) and the gene expression data to operate the univariate Cox regression analysis and the Least absolute shrinkage and selection operator (LASSO) regression by “survival” 26. We further obtained 6 survival-related BMGs. After the multivariate Cox regression analysis, we finally reserved 3 significantly differential BMGs that were strongly linked with the prognosis and survival of AML samples.

### Risk score calculation and nomogram construction

The risk score was calculated based on the expression levels and relevant coefficient of the 3 BMGs, and the formula was as follows: the risk score = Coef1*Exp1 + Coef2*Exp2 + Coef3*Exp3. Then, we divided the training cohort into high- and low-risk groups based on median value. To assess survival of high- and low-risk groups, we performed Kaplan-Meier (K-M) analysis by using the “survminer” package. To test its accuracy, we plotted 1-, 3-, and 5-year Receiver Operating Characteristic (ROC) curves by using “timeROC” package 27. To examine the ability of risk score to distinguish different samples, we plotted ranked dot and scatter plots using the “ConsensusClusterPlus” package 28. Meanwhile, we also used testing cohort (GSE12417 and GSE37642) to validate the predictive efficiency of risk score. We also performed K-M analysis, and plotted the ranked dot and scatter plots and ROC curve, respectively.

Based on the risk score and clinicopathological features, we constructed the nomogram model and calibration curve and calculated the area under curve (AUC) and the 1-, 3-, and 5- year survival rate to predict the prognosis by using the “survival” packages.

### The clustering analysis of BMGs

We used the “ConsensusClusterPlus” package to divide the training cohort into two clusters (cluster A and B) based on BMGs. Then, we used "survival" package to analyze the K-M survival difference between the two clusters. To test whether the samples of BMGs can effectively distinguish the clustering results, we used the “ConsensusClusterPlus” packages to draw PCA plots. In addition, we plotted the Sankey's diagram by using the “ggalluvial” and “dplyr” packages 29 to show the relationship among clusters, risk groups, and survival status.

To determine the relationship between biological behaviors and different gene clusters, we downloaded “c2.cp.kegg.v7.4.symbols.gmt” from MSigDB. The heatmap for displaying the differential pathways was plotted by using “GSEABaes” and “GSVA” packages 30, 31. To investigate the association of gene clusters with the tumor microenvironment (TME) and to evaluate the levels of immune cell infiltration, we performed single-sample gene set enrichment analysis (ssGSEA) using “GSEAbase” and “GSVA” packages.

We used the “limma” packages to screen the differential genes of the training cohort based on gene clusters. The screening criteria were the adjusted p <0.05 and log|Fc|>1. In addition, to analyze the potential biological functions and signaling pathways that may be involved, Gene Ontology (GO) and Kyoto Encyclopedia of Gene and Genomes (KEGG) enrichment analyses were performed by using “clusterProfiler” packages 31.

### Immune evaluation of TME between the high- and low-risk groups

To study the related immune cells of BMGs, the proportion of immune cells was quantified based on risk score by using the CIBERSORT algorithm. The relative scatters diagram and heatmap were plotted by using “ConsensusClusterPlus” package 32. The AML samples in the fraction of 23 immune subsets were calculated with the CIBERSORT algorithm 33. To explore the tumor purity in the TME, the score was estimated by the “ESTIMATE” package 34.

### The analysis of mutation and drug sensitivity in AML

We summarized the frequency and somatic mutation of CNV and plotted the landscape of genetic alternation and expression variation. Next, we explored the drug sensitivity between the high- and low-risk groups by “pRRophetic” packages 35.

## Results

### The genetic landscape of BMGs in AML

**Figure 1:**
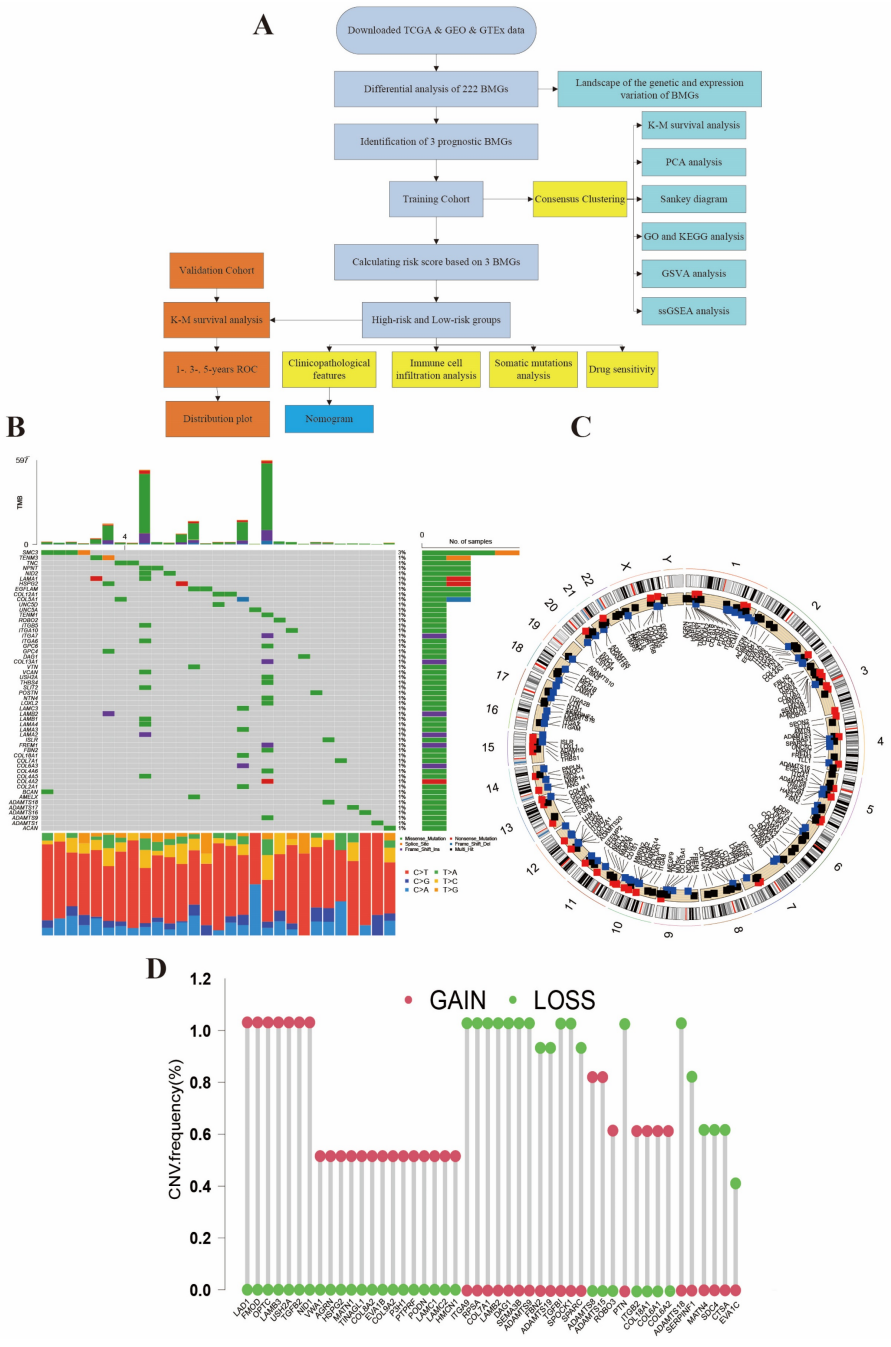
Genetic mutation of BMGs in AML. (A) The flow chart of this study. (B) Tumor mutation burden frequencies of 222 BMGs in 134 AML patients. (C) Locations of the CNV alteration on chromosomes in BMGs. (D) Frequencies of CNV amplification and deletion among major BMGs.

We performed preliminary processing of the data from public database and summarized all samples' clinical features in Table 1. The main process of this study is shown in Figure 1A. Figure 1B shows the genetic landscape of BMGs in AML and the genetic mutation frequency of 222 genes. Among 134 samples, mutations were detected in 29 (21.64%) AML samples. Among mutation genes, SMC3 was the gene with the highest mutation rate (3%). Expected SMC3, the other 53 genes all had 1% mutation rate. Figure 1C displays the chromosome location of CNV alteration of BM-related genes. Meanwhile, in the CNV analysis, we found frequent alternations in BMGs (Figure 1D and Figure S1). The CNV amplifications are shown in red spots and the deletions are shown in green spots. LAD1, FMOD, OPTC, LAMB3, USH2A, TFGB2, NID1, ADAMTS8, ADAMTS15, ROBO3, ITGB2, COL18A1, COL6A1, and, COL6A2 were the most amplified BMGs, while ITGA9, RPSA, COL7A1, LAMB2, DAG1, SEMA3B, ADAMTS9, FBN2, ADAMTS19, TGFBI, SPOCK1, SPARC, PTN, ADAMTS18, SERPINF1, MATN4, SDC4, and CTSA had the most deletion mutations.

### Identification of the differential BMGs and division of the high- and low-risk groups

**Figure 2:**
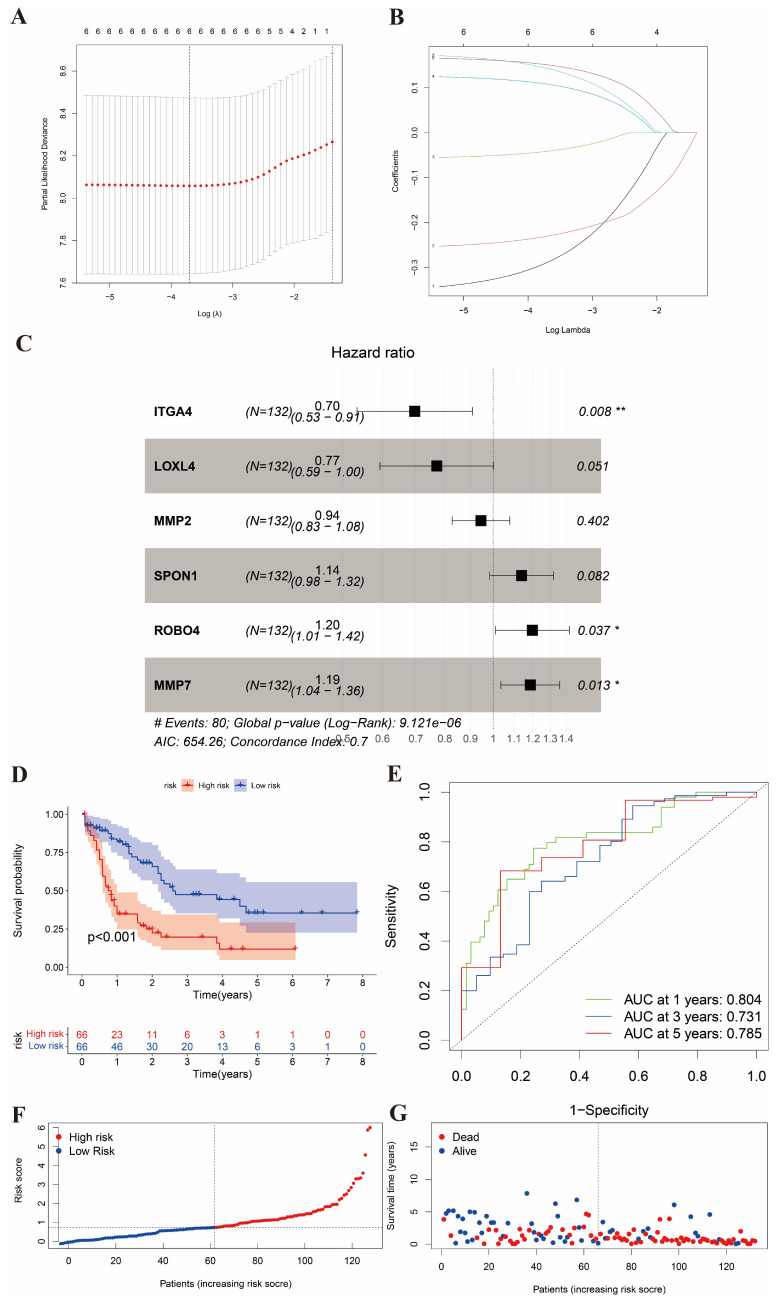
Identification of the differential BMGs and division of two risk groups. (A-B) LASSO analysis for 6 genes. (C) Hazard ratio of multivariate Cox model to identify 3 BMGs. (D) K-M analysis of survival in high- and low-risk groups. (E) ROC with AUC at 1-, 3-, and 5-years. (F-G) Risk curve and survival status curve showing risk score distribution.

488 AML and normal samples were obtained from TCGA. According to the criteria of log|Fc|>2 and P value < 0.05, we identified 20 differential BMGs from 222 BMGs. By univariate Cox regression analysis and LASSO, 6 differential genes with the optimal λ value were obtained (Figure 2A and 2B). Three differential genes (ITGA4, ROBO4, and MMP7) were eventually identified as the prognostic BMGs after multivariate Cox regression analysis (Figure 2C). It showed that ITGA4 was downregulated and other 2 genes were upregulated in AML. In addition, we preserved the coefficients of 3 prognostic BMGs to calculate the risk score (Table S2).

We divided the training cohort into high- and low-risk groups based on the median risk score calculated from the expression levels of the 3 BMGs, which reflects the stratification solely by risk score without considering transcriptional patterns (Figure 2D). The patients with low scores had a distinctly favorable overall survival compared to those with high scores. In addition, the AUC of 1-, 3-, and 5-year ROC curves was 0.804, 0.731, and 0.785, respectively (Figure 2E). Moreover, the distribution plot of the risk score displayed that the survival time decreased with the increase of the risk score (Figure 2F and 2G).

### The evaluation of risk score in validation cohorts

**Figure 3:**
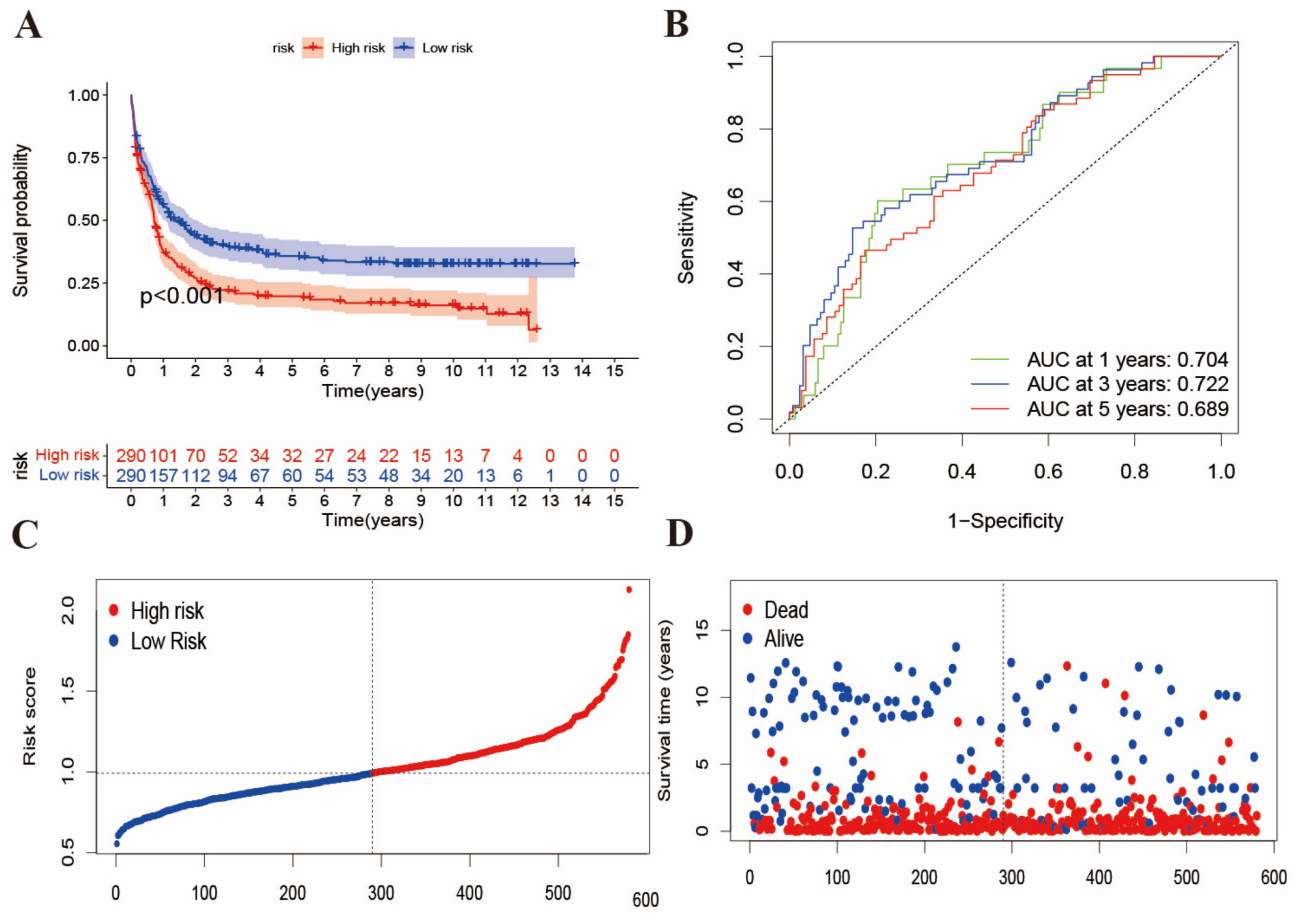
The evaluation of risk score in the validation cohort. (A) K-M analysis of survival in external validation cohort. (B) ROC curves to predict the sensitivity and specificity of 1-, 3-, and 5-year survival of validation cohort. (C-D) risk curve and survival status curve showing risk score distribution in validation cohort.

To validate the BMGs predictive performance, we introduced two external datasets (GSE12417 and GSE37642) as the validation cohort. First, we merged the samples from two datasets and obtained 585 samples. The patients were also divided into high- and low-risk groups according to the same methods of the training cohort. Then, we performed survival analysis and plotted the ROC curve, and risk score distributed plots, respectively (Figure 3A-3D). In Figure 3A, K-M analysis showed that the two groups had great survival difference (P<0.001). Moreover, we mapped the ROC to evaluate the prognostic values (Figure 3B). In addition, Figure 3C-3D reveals that the risk curve and survival status curve of four cohorts suggested that the high-risk sample can be easily separated from other samples. Combined with Figure 2E, the validation cohort AUCs of 1-, 3- and 5 years were 0.704, 0.722, and 0.689, respectively. Above all, we found that the risk score could be perfectly used to predict the clinical outcome of AML patients.

### Construction of a nomogram model to predict prognosis

**Figure 4:**
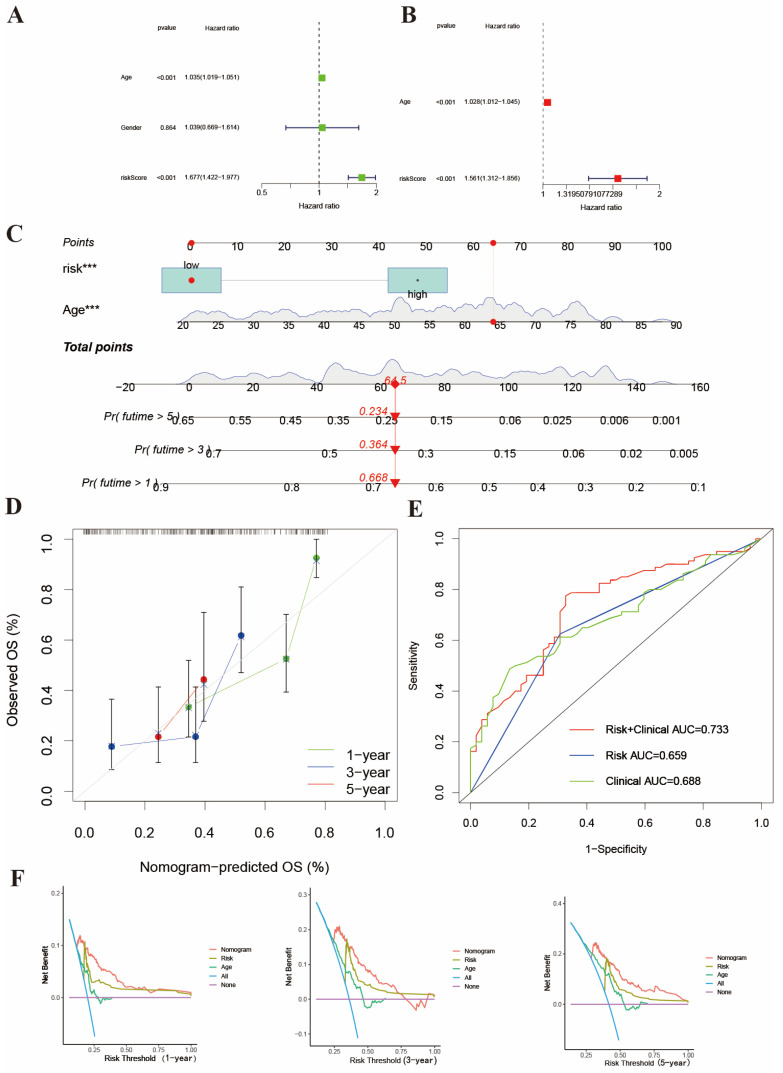
Construction of nomogram to predict the survival of AML patients. (A-B) Univariant and multivariant Cox analysis of clinicopathological and risk score of AML samples. (C) The nomogram integrates age and risk score for AML patients. (D) 1-, 3- and 5-years calibration curves of nomogram. (E) AUC of nomogram, risk score and age. (F) The DCA curves of the nomograms compared for 1-, 3- and 5-years overall survival in AML.

We constructed a nomogram model based on risk score and clinicopathological features (Age). We finally identified age and risk score as independent prognostic factors to establish a nomogram after univariate and multivariant Cox regression analysis (Figure 4A-4B). This model could predict the 1-, 3- and 5-year survival rates of AML patients (Figure 4C). Meanwhile, the calibration curve is shown in Figure 4D. The integrated AUC (Figure 4E) in predicting the matched degrees between high-risk patients and death outcome was 0.733, which was significantly higher than age (AUC=0.688) and risk score (AUC=0.659). In Figure 4F, DCA analysis also shown this exciting result. The results suggest that the nomogram model may have stronger efficiency to predict the prognosis of AML patients and that high-risk AML patients usually accompanied with poor survival outcomes.

### Identification of gene clusters based on 3 BMGs

**Figure 5:**
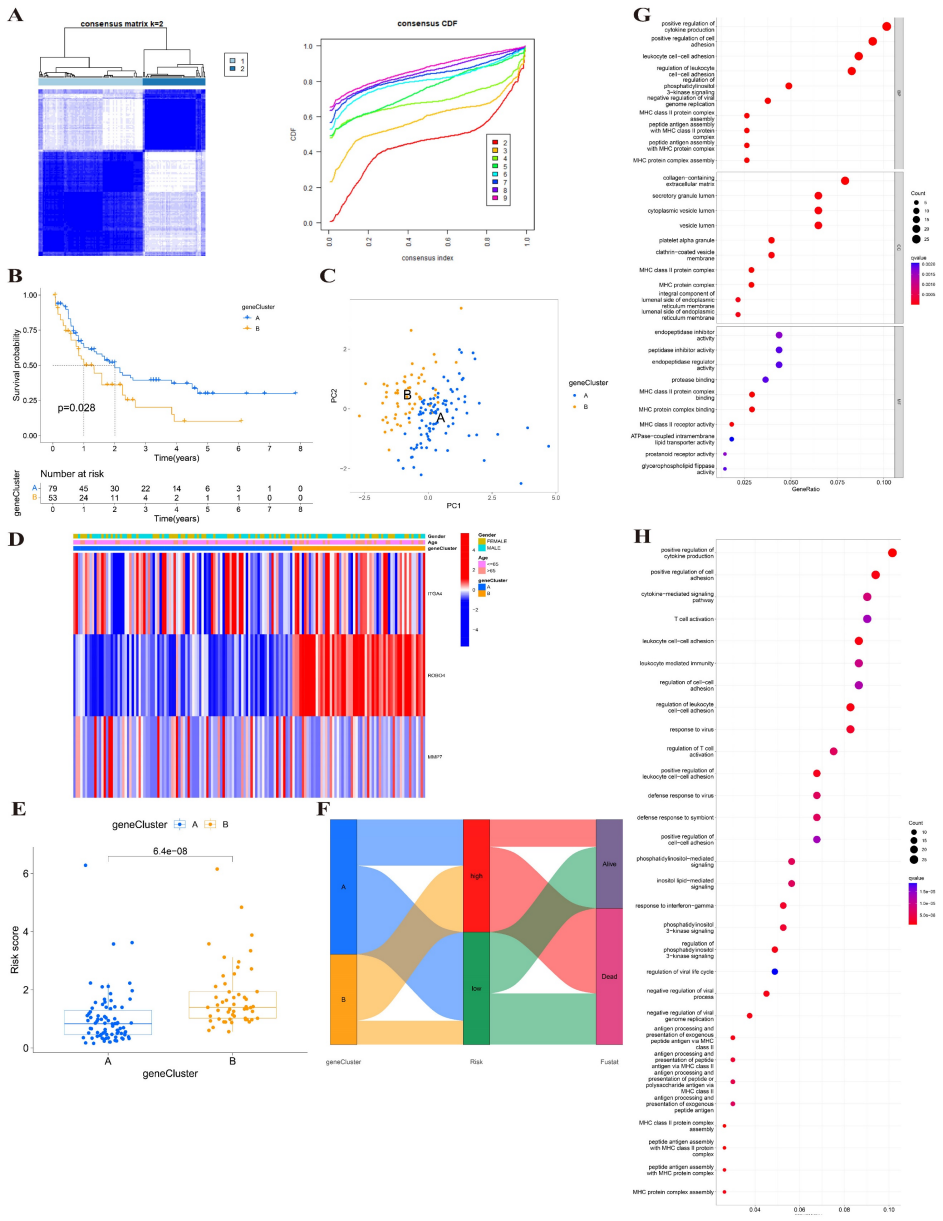
Identification of BMGs gene clusters. (A) Consensus heatmap of two clusters (k=2). (B) K-M analysis showing the relationship of 3 BMGs with the survival time. (C) PCA analysis between two gene clusters. (D) Heatmap of gene clusters and clinicopathological features of AML patients. (E) The relationship of risk score and gene clusters (p<0.001). (F) Sankey's diagram of gene cluster distribution among high- and low-risk groups and clinical outcomes. (G-H) GO and KEGG enrichment analysis between two gene clusters.

Using the transcriptional profiles of the 3 BMGs, we applied a consensus clustering algorithm to stratify the training cohort into two clusters, designated as cluster A and cluster B (Figure 5A). These clusters exhibited distinct transcriptional patterns, and cluster B was associated with a higher risk score and poorer prognosis (Figure 5B). The PCA analysis of the two clusters showed that the two clusters could greatly distinguish two subtypes (Figure 5C). The relationship between two gene clusters with clinical characteristics was shown in Figure 5D. There was a distinct difference in risk score between two gene clusters. The risk score of cluster B was higher than cluster A (p=6.4e-08) (Figure 5E). In addition, Sankey's diagram was used to visualize the relationship among clusters, risk groups, and survival status (Figure 5F). Therefore, cluster B was associated with high-risk group and poor outcome.

We screen the differential genes of the training cohort based on distinct gene subtypes. We identified 285 differential genes between two subtypes and then analyzed the biological functions and pathways of these genes by using GO and KEGG enrichment analysis. GO enrichment analysis showed that these genes were significantly enriched in cellular functions such as cellular adhesion and regulation (Figure 5G). The KEGG pathway enrichment analysis of differential genes displayed that they mainly participated in cellular activities and regulative pathways (Figure 5H).

### The features of the biological activities and TME between two clusters

**Figure 6:**
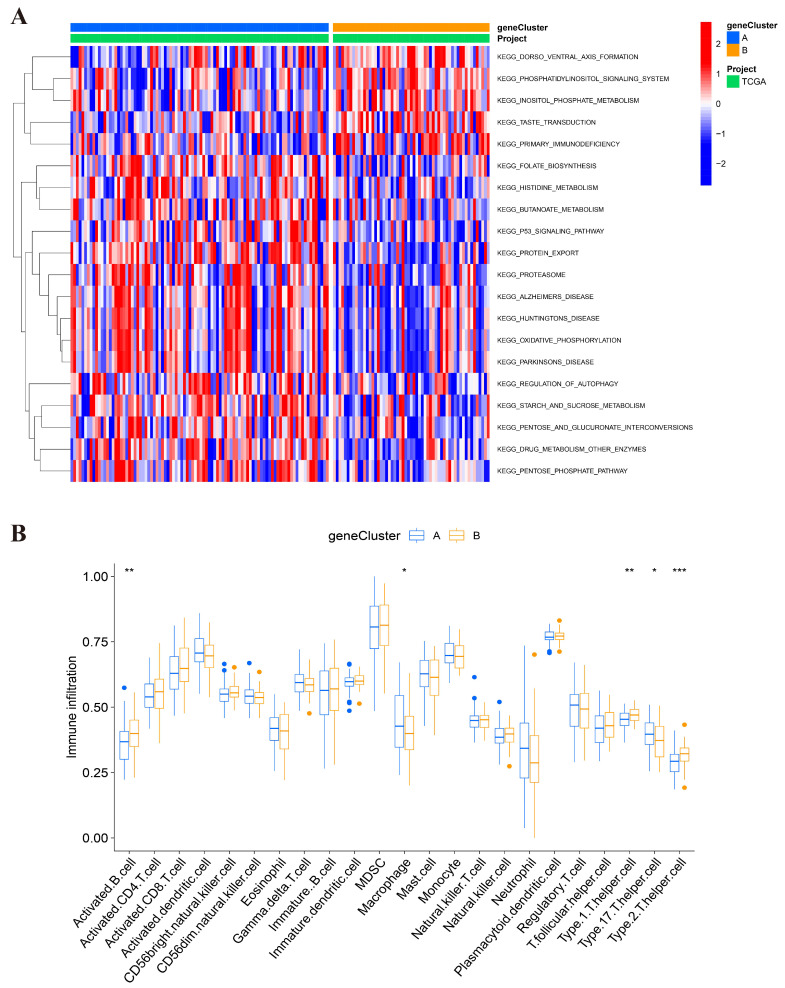
Analysis between two clusters. (A) GSVA analysis of biological enrichment pathways in two gene clusters. (B) ssGSEA analysis of infiltrating immune cell types in two clusters.

To determine different biological behaviors of distinct gene clusters, we performed GSVA analysis and plotted a heatmap. As shown in Figure 6A, Cluster A showed enrichment in protein metabolism and protein-relative diseases, such as protein export, proteasome, regulation of autophagy, drug metabolism and enzymes, pentose phosphate pathway, Alzheimer's disease, Parkinson's disease, and Huntington's disease. Another main metabolism pathway was glycometabolism, including starch and sucrose metabolism, and the interconversion of pentose and glucuronate. This indicates that cluster A may be more likely to be associated with significant biochemistry reactivity, especially protein function and glycometabolism. Cluster B showed significant enrichment of the intercellular and intracellular signal transduction and their related pathways, such as dorsoventral axis formation, phosphatidylinositol signaling system, inositol phosphate metabolism, and taste transduction.

We performed ssGSEA analysis to investigate the relationship between immune cells and gene clusters and to explore the role of BMGs in the TME of AML. The cell infiltration was analyzed and the activated immune cells were identified (Figure 6B). Cluster A was only associated with higher immune infiltration of macrophages. However, cluster B was related to the upregulation of activated B cells, and type1, 2, 17 Th. Thus, the infiltrated immune cells in cluster B may be associated with poor prognosis.

### The immune infiltration in the high- and low-risk group

**Figure 7:**
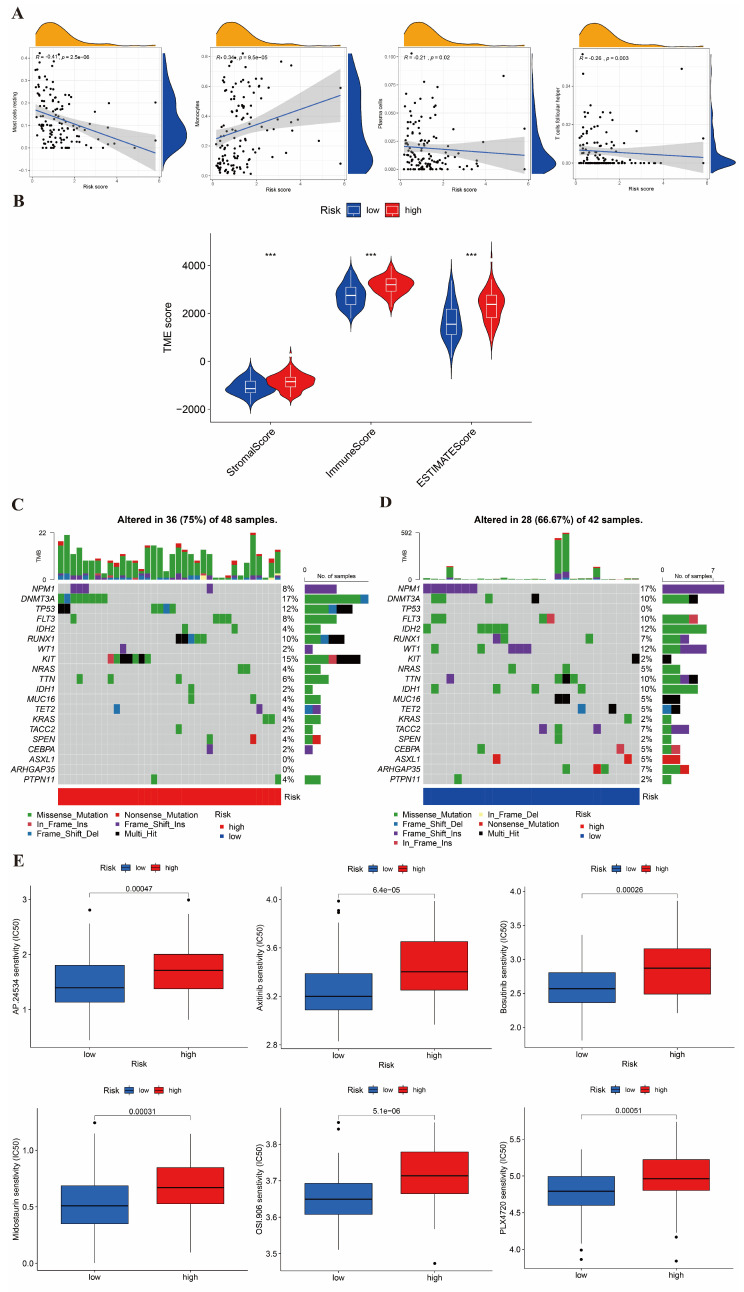
Estimation of the TME, somatic mutation frequencies, and drug sensitivity between high- and low-risk groups. (A) Relationship between risk score and immune cell type. (B) Relationship between high- and low-risk groups and TME score. (C-D) Frequencies of CNV between high- and low-risk groups. (E) The relationship between the high-risk group and sensitive drugs.

We also evaluated the relationship between the risk score and the abundance of immune cells using the ESTIMATE algorithm. In Figure 7A, the ESTIMATE score was significantly associated with the abundance of resting mast cell (cor=-0.41), monocyte cell (cor=0.34), plasma cell (cor=-0.21), and follicular helper T cell (cor=-0.26). In addition, we also assessed the association between high-/low-risk groups and the ESTIMATE scores (Figure 7B), which showed that a high- risk score was also related to a higher immune score, stromal score, and estimate score.

### The mutation and drug sensitivity analysis in AML

We analyzed the CNV of the somatic mutations between high- and low-risk groups in the TCGA training cohort. According to our previous risk score model, we found the CNV genes and mutation classifications of high- and low-risk groups. The mutations of DNMT3A, KIT, TP53, and RUNX1 were shown in Figure 7C, with the highest mutation rate in the high-risk group. The main mutation forms of DNMT3A were missense mutation and frameshift deletion; those of KIT were missense mutation, nonsense mutation and frame hit; those of TP53 were missense mutation, multi-hit, and frameshift deletion; and, those of RUNX1 were missense mutation, multiple hits, and frameshift deletion. In Figure 7D, it is shown that NPM1, IDH2, DNMT3A, WT1, and IDH1 were the five genes with the highest mutation rate in the low-risk group. The mutation forms of NPM1 were frameshift insertion; those of IDH1 and IDH2 were missense mutation; those of WT1 were missense mutation and frameshift insertion; and, those of DNMT3A were missense mutation and multiple hits.

Finally, we estimated the sensitivities of patients to current therapeutic drugs in the two risk groups. To analyze the drug sensitivity of AML cells to the small molecular drugs, we used the IC50 data of drugs from "pRRophetic" package. Moreover, we obtained the 15 differential results of drug sensitivity in two risk groups (Figure 7E and Figure S2). We found that the high-risk group had a lower IC50 values of Roscovitine and Bortezomib; while, the low-risk group had a lower IC50 value of Axitinib, Bosutinib, Midostaurin, Thapsigargin, and AKT inhibitor VIII.

## Discussion

In this study, we identified three BMGs through bioinformatics analysis and established a risk score model to predict the prognosis of AML patients. While most previous studies on BMGs have focused on their roles in solid tumors, our findings expand their significance to hematological malignancies, particularly AML. Unlike solid tumors, where the BM acts as a barrier that tumor cells must breach for metastasis, AML originates and progresses within the unique hematopoietic bone marrow microenvironment. This niche provides structural and functional support for hematopoietic stem and progenitor cells and plays a pivotal role in leukemogenesis.

ROBO4 and MMP7, two of the key BMGs identified in our study, are extensively documented for their involvement in angiogenesis and ECM remodeling in solid tumors. We propose that their dysregulation may contribute to AML leukemogenesis through mechanisms specific to the bone marrow microenvironment, such as disrupting ECM integrity and altering angiogenesis within the niche. ROBO4, as a critical regulator of vascular stability and angiogenesis, may lead to aberrant angiogenesis and increased vascular permeability when dysregulated. This could disrupt the bone marrow niche and create a permissive environment for leukemic proliferation 36, 37. Similarly, MMP7's proteolytic activity may facilitate ECM degradation, disrupting cellular adhesion and promoting the mobilization of leukemic cells 22, 24. This degradation may also disrupt cytokine gradients and immune interactions within the bone marrow niche, contributing to leukemic expansion. Dysregulated ROBO4 expression can lead to aberrant angiogenesis and increased vascular permeability, potentially disrupting the bone marrow niche and creating a permissive environment for leukemic proliferation 22.

We established an effective risk score model based on 3 BMGs to predict prognosis. We performed analyses on clinicopathological characteristics, prognosis, mutation, TME, and drug sensitivity and found significant differences between the two risk groups. Moreover, based on risk scores and patients' age, we conducted a nomogram model and a calibration curve. We found that BMGs may predict the clinical and therapeutic outcomes of AML. This model might be used to classify AML patients and provide novel ideas for targeted therapies.

In our study, Sankey's diagram indicated that cluster A and B were associated with low- or high-risk groups. To further explore whether the specific signaling of gene clusters are different in AML, we performed GO and KEGG analysis. We found that the mainly enriched pathways included cellular or intercellular adhesion and the regulation of cytokine production. Therefore, we assume that cellular adhesion is closely related to the progression of the tumor. Next, we investigated the relationship among cellular adhesion molecules, cytokine regulation, and AML. Cellular adhesion molecules have been widely studied in many solid tumors. Focal adhesion kinase (FAK) promoted CD8+ T cell depletion and Treg recruitment by regulating chemokine or cytokine transcription, which suppressed immune reactivities and promoted squamous cell carcinoma survival 38. In colon cancer, FAK phosphorylation enhanced the activity of the transcription factor NANOG, which activated PTK2 to initiate the NK-κB pathway to support the progression of carcinoma cells. For another, NANOG also promoted the expression of FAK in colon carcinoma cells through positive feedback 39. Moreover, the inhibitor of FAK can make pancreatic cancer sensitive to checkpoint therapy again 40. In recent study, FAK supported the survival of AML cells by regulating the interaction between leukemia and stromal 41. In addition, other cell adhesion molecules, such as CD44 and CD56, be associated with CXCL12-induced chemoresistance and the promotion of AML progression 42, 43. As for cytokine, IL10 inhibits cytokine production of activated macrophages and T-helper 1 cells and help AML cell to escape 44. Survivin family expression and regulation can be detected and played an important role in suppressing apoptosis in AML 45. Finally, other cytokines, such as TNF-α, IL-1β, and IL-6 tended to increase the aggressiveness of AML, and anti-inflammatory molecules such as TGF-β seem to block AML progression. Dysregulation of the complicated interactions between proinflammatory and anti-inflammatory cytokines in AML may create a tumor-promoting microenvironment that influences the proliferation, survival, and drug resistance of AML cells 46.

TME, in which immune cells (such as monocytes, neutrophils, lymphocytes, and macrophages) are the main components, is importantly involved in AML progression. These immune cells can participate in various immune reactivities and inflammatory responses to assist in tumor survival 47. The ssGSEA analysis showed cluster B with poor prognosis more infiltrate Th1 and Th 2 cells whereas Th17 was linked with a better prognosis, which revealed the roles of Th cells in AML. Th1/Th2 imbalance is involved in the autophagy and development of AML. IRF2 - INPP4B axis has been shown to inhibit apoptosis by inducing autophagy in AML. And IRF2-INPP4B axis mediated regulation of Th1/Th2 balance has promoted autophagy and inhibited apoptosis in AML 48. However, Th17 cells actively suppressed the immune state and may promote infections and probably tumor escape 49. T follicular helper cells can increase the expression of chemokine receptor CXCR5, while decreasing the expression of CCR7, and migrating to B cell zone under the action of chemokine CXCL13 produced by stromal cells in B cell zone. The interaction between T follicular helper cells and B cells can promote immune-activated germinal center response, which further activates CCL19-21/CCR7 axis and CXCL13/CXCR5 axis and promotes tumor development 50-52. In AML, these mechanisms may explain that poor prognosis is relevant to high infiltration of cluster B and unveiled the complex roles of immune cells in tumors.

In addition, our results provide a foundation for future investigations into the functional consequences of ROBO4 and MMP7 dysregulation in AML. Clinical trials targeting angiogenesis and ECM components in AML, such as those focusing on VEGF inhibitors and matrix metalloproteinase inhibitors, could benefit from incorporating BMG profiling to stratify patient subgroups and optimize therapeutic outcomes 53.

However, there are some limitations in this study. Firstly, all analyses were based on data downloaded from public databases, and all samples used in our study were obtained retrospectively. Thus, selective bias is inevitable. More independent AML datasets should be used in the future to improve the accuracy of prognostic models. Large prospective studies and additional *in vivo* and *in vitro* studies are needed to verify our findings. In addition, other clinical data, like gender, and stages, are unavailable, which may affect the prediction of prognosis.

## Conclusion

We identified 3 BMGs by using bioinformatics and constructed a prognostic model to predict the survival of AML patients. Our study revealed that BMGs played an important role in immune TME. Finally, this study confirmed that BMGs were related to AML development. Our findings provide new ideas for guiding therapy for patients with AML.

## Supplementary Material

Supplementary figures and tables.

## Figures and Tables

**Table 1:** The clinical characteristics of studied datasets.

	GSE37642	TCGA AML	GSE12417
Sample counts	402	142	162
Age, years mean (SD)	54.57(14.90)	54.39(16.34)	55.63(14.88)
Gender Male	-	78	-
Female	-	64	-
FAB subtypes M0	14	-	5
M1	84	-	45
M2	117	-	45
M3	19	-	0
M4	104	-	42
M5	47	-	19
M6	15	-	6
M7	2	-	0
Overall survival Time, years mean (SD)	2.84(3.74)	1.57(1.64)	1.25(1.16)
Survival status Dead	295	89	103
Alive	107	53	59
