# Supplementary material for: Establishment of a Basement Membrane-Related Prognosis Model and Characterization of Tumor Microenvironment Infiltration in Acute Myeloid Leukemia
Source: J Cancer. 2025 Jan 13;16(4):1228–42. doi: 10.7150/jca.108041 (PMC11786050; doi:10.7150/jca.108041)
Supplement: Supplementary file 1 — Supplementary figures and tables. [file jcav16p1228s1.pdf]

Supplementary materials

Supplementary Figure S1 Frequencies of CNV amplification and deletion among 222 BMGs.
Supplementary Figure S2 The relationship between high-risk and low-risk groups and sensitive drugs.
Supplementary Table S1 The list of 222 BMGs from previously published articles.
Supplementary Table S2 The coefficient of 3 BMGs after multivariate Cox regression analysis.

S1

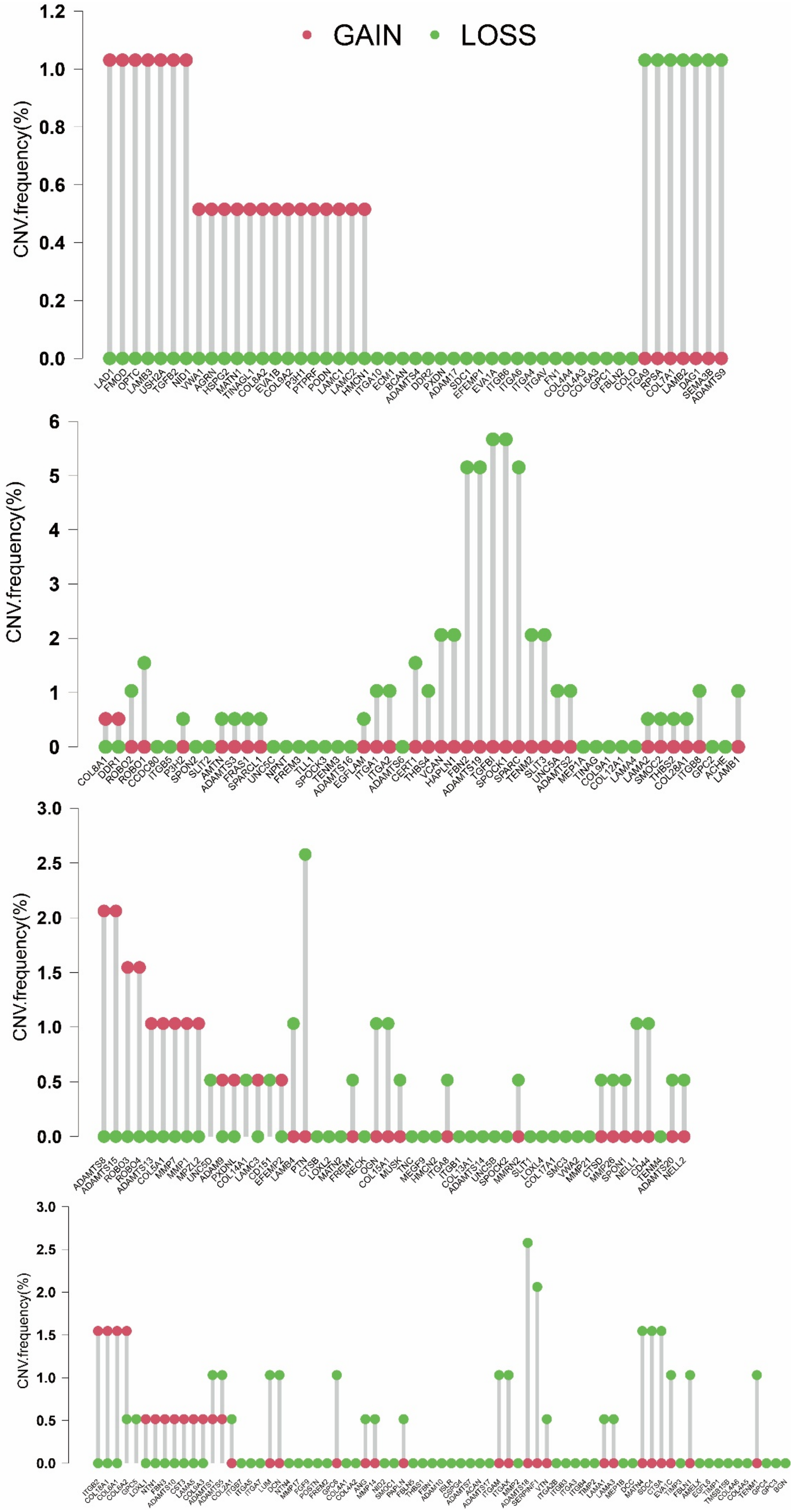

S2

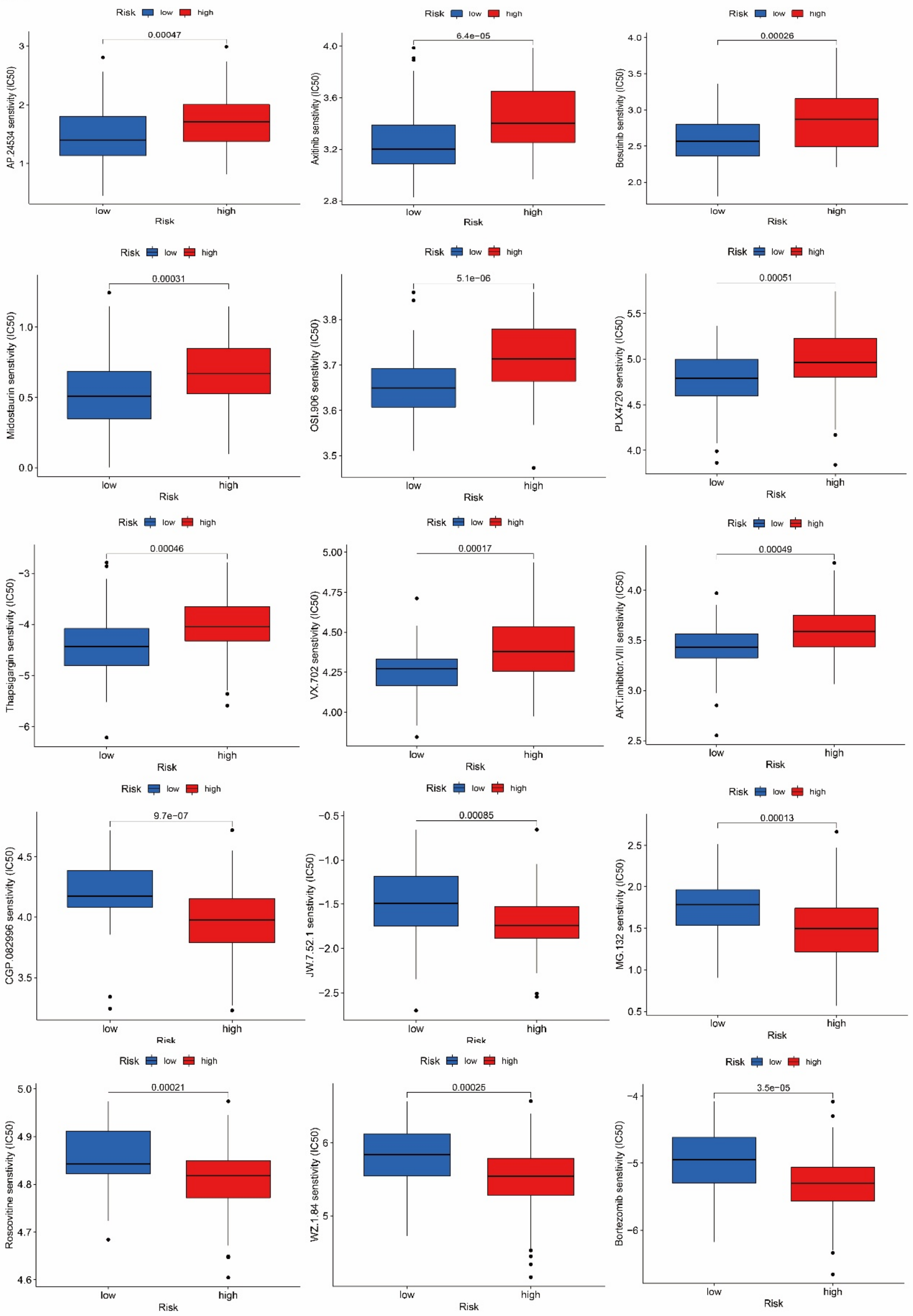

Risk low high
0.00047
AP 24534 sensitivity (IC50)
6.4e−05
Axitinib sensitivity (IC50)
0.00026
Bosutinib sensitivity (IC50)
0.00031
Midostaurin sensitivity (IC50)
5.1e−06
OSI.906 sensitivity (IC50)
0.00051
PLX4720 sensitivity (IC50)
0.00046
Thapsigargin sensitivity (IC50)
0.00017
VX.702 sensitivity (IC50)
0.00049
AKT.inhibitor.VIII sensitivity (IC50)
9.7e−07
CGP.082996 sensitivity (IC50)
0.00085
JW.7.52.1 sensitivity (IC50)
0.00013
MG.132 sensitivity (IC50)
0.00021
Roscovitine sensitivity (IC50)
0.00025
WZ.1.84 sensitivity (IC50)
3.5e−05
Bortezomib sensitivity (IC50)
low
high
Risk

Supplementary Table S1 The list of 222 BMGs from previously published articles.

Gene	Type
ACAN	BM
ACHE	BM
ADAMTS1	BM
ADAMTS2	BM
ADAMTS3	BM
ADAMTS4	BM
ADAMTS5	BM
ADAMTS6	BM
ADAMTS7	BM
ADAMTS8	BM
ADAMTS9	BM
ADAMTS10	BM
ADAMTS13	BM
ADAMTS14	BM
ADAMTS15	BM
ADAMTS16	BM
ADAMTS17	BM
ADAMTS18	BM
ADAMTS19	BM
ADAMTS20	BM
AGRN	BM
AMELX	BM
AMTN	BM
ANG	BM
BCAN	BM
BGN	BM
CCDC80	BM
CERT1	BM
COL2A1	BM
COL4A1	BM
COL4A2	BM
COL4A3	BM
COL4A4	BM
COL4A5	BM
COL4A6	BM
COL5A1	BM
COL6A1	BM
COL6A2	BM
COL6A3	BM
COL7A1	BM
COL8A1	BM

COL8A2	BM
COL9A1	BM
COL9A2	BM
COL9A3	BM
COL12A1	BM
COL14A1	BM
COL15A1	BM
COL17A1	BM
COL18A1	BM
COL28A1	BM
COLQ	BM
CST3	BM
CTSA	BM
CTSB	BM
CTSD	BM
DCN	BM
ECM1	BM
EFEMP1	BM
EFEMP2	BM
EGFL6	BM
EGFLAM	BM
FBLN1	BM
FBLN2	BM
FBLN5	BM
FBN1	BM
FBN2	BM
FBN3	BM
FGF9	BM
FMOD	BM
FN1	BM
FRAS1	BM
FREM1	BM
FREM2	BM
FREM3	BM
HAPLN1	BM
HMCN1	BM
HMCN2	BM
HSPG2	BM
ISLR	BM
LAD1	BM
LAMA1	BM
LAMA2	BM
LAMA3	BM

LAMA4	BM
LAMA5	BM
LAMB1	BM
LAMB2	BM
LAMB3	BM
LAMB4	BM
LAMC1	BM
LAMC2	BM
LAMC3	BM
LOXL1	BM
LOXL2	BM
LOXL4	BM
LUM	BM
MATN1	BM
MATN2	BM
MATN4	BM
MEP1A	BM
MEP1B	BM
MMP1	BM
MMP2	BM
MMP7	BM
MMP17	BM
MMP21	BM
MMP26	BM
MMRN2	BM
NELL1	BM
NELL2	BM
NID1	BM
NID2	BM
NPNT	BM
NTN1	BM
NTN4	BM
OGN	BM
OPTC	BM
P3H1	BM
P3H2	BM
PAPLN	BM
PODN	BM
POSTN	BM
PTN	BM
PXDN	BM
PXDNL	BM
RECK	BM

SERPINF1	BM
SLIT1	BM
SLIT2	BM
SLIT3	BM
SEMA3B	BM
SMC3	BM
SMOC1	BM
SMOC2	BM
SPARC	BM
SPARCL1	BM
SPOCK1	BM
SPOCK2	BM
SPOCK3	BM
SPON1	BM
SPON2	BM
TGFB2	BM
TGFB1	BM
TGFBI	BM
THBS1	BM
THBS2	BM
THBS4	BM
TIMP1	BM
TIMP2	BM
TIMP3	BM
TINAG	BM
TINAGL1	BM
TLL1	BM
TNC	BM
USH2A	BM
VCAN	BM
VTN	BM
VWA1	BM
VWA2	BM
ADAM9	BM
ADAM10	BM
ADAM17	BM
CD44	BM
CD151	BM
CSPG4	BM
COL13A1	BM
DAG1	BM
DCC	BM
DDR1	BM

DDR2	BM
EVA1A	BM
EVA1B	BM
EVA1C	BM
GPC1	BM
GPC2	BM
GPC3	BM
GPC4	BM
GPC5	BM
GPC6	BM
ITGA1	BM
ITGA2	BM
ITGA2B	BM
ITGA3	BM
ITGA4	BM
ITGA5	BM
ITGA6	BM
ITGA7	BM
ITGA8	BM
ITGA9	BM
ITGA10	BM
ITGAM	BM
ITGAV	BM
ITGAX	BM
ITGB1	BM
ITGB2	BM
ITGB3	BM
ITGB4	BM
ITGB5	BM
ITGB6	BM
ITGB7	BM
ITGB8	BM
MEGF9	BM
MMP14	BM
MPZL2	BM
MUSK	BM
PTPRF	BM
RPSA	BM
ROBO1	BM
ROBO2	BM
ROBO3	BM
ROBO4	BM
SDC1	BM

SDC4	BM
TENM1	BM
TENM2	BM
TENM3	BM
TENM4	BM
UNC5A	BM
UNC5B	BM
UNC5C	BM
UNC5D	BM

Supplementary Table S2 The coefficient of 3 BMGs after multivariate Cox regression analysis.

	coef	exp(coef)	se(coef)	z	Pr(>|z|)
ITGA4	-0.36235	0.69604	0.13555	-2.673	0.00751 **
ROBO4	0.18048	1.1978	0.08667	2.082	0.03730 *
MMP7	0.17077	1.18622	0.06912	2.471	0.01349 *
Signif. codes: '***' 0.001, '**' 0.01, '*' 0.05					
